# Spontaneous dural tear leading to intracranial hypotension and tonsillar herniation in Marfan syndrome: a case report

**DOI:** 10.1186/1471-2377-10-54

**Published:** 2010-06-28

**Authors:** Aqueel H Pabaney, Farhan A Mirza, Nadir A Syed, Humera Ahsan

**Affiliations:** 1 Department of Neurology, Aga Khan University Hospital, Stadium Road, P.O. Box 3500, Karachi, Pakistan; 2 Medical College, Aga Khan University Hospital, Stadium Road, P.O. Box 3500, Karachi, Pakistan; 3 Department of Neuroradiology, Aga Khan University Hospital, Stadium Road, P.O. Box 3500, Karachi, Pakistan

## Abstract

**Background:**

We describe the case of a 38 year old male with Marfan syndrome who presented with orthostatic headaches and seizures.

**Case Presentation:**

The patient was diagnosed with Spontaneous Intracranial Hypotension secondary to CSF leaks, objectively demonstrated by MR Myelogram with intrathecal contrast. Epidural autologus blood patch was administered at the leakage site leading to significant improvement.

**Conclusion:**

Our literature search shows that this is the second reported case of a Marfan patient presenting with symptomatic spontaneous CSF leaks along with tonsillar herniation.

## Background

Spontaneous intracranial hypotension (SIH) is an under diagnosed entity that was first described by Schaltenbrand in 1938 [1]; however, a more objective description of SIH was later proposed as a "decrease in CSF pressure to less than 60 mm H2O associated with occipital headaches [2,3]". An emergency department based study estimated the annual incidence of SIH to be 5 per 100,000 [4]; this condition is twice as common in females [5].

A traumatic event and generalized connective tissue disorders (CTDs) are amongst the commonest etiologies for SIH. Among the CTDs, Marfan,[6-8] Ehlers-Danlos type II [9] and autosomal dominant polycystic kidney disease [10] are associated with spontaneous CSF leaks leading to SIH.

SIH can present in a variety of ways including *orthostatic *headache, diplopia, tinnitus, photophobia, hyperacusis and vomiting [11-13]. On clinical examination, bradycardia, nystagmus, Abducens nerve palsy and neck stiffness are common findings [14,15]. Radiologically, there are 5 characteristic features of SIH on MR imaging: 1. **S**ubdural fluid collections; 2.**E**nhancement of pachymeninges; 3. **E**ngorgement of venous structures; 4. **P**ituitary hyperemia; 5. **S**agging of the brain (acronym: **SEEPS**) [16]. MR Myelography with intrathecal contrast is considered the imaging modality of choice to accurately locate the CSF leak [3].

We present a case of intracranial hypotension secondary to a spontaneous dural tear in an adult patient with Marfan syndrome.

## Case Presentation

Our patient, a 38 year old male, diagnosed case of Marfan's syndrome, presented with complaints of orthostatic headaches and one episode of seizure. His current illness started 15 days ago with bilateral neck pain, which progressed to a holocranial headache which would get markedly worse on sitting, standing or bending forward and relived by lying down. These symptoms progressed over the past 2 weeks and on the day of presentation he suffered a generalized tonic - clonic seizure which prompted admission. There was no history of trauma.

On examination, our patient was hemodynamically stable and afebrile. He displayed characteristic marfanoid features including micrognathia, tall and lean stature, disproportionately long limbs and joint hypermobility. On detailed neurological examination, he was drowsy but arousable to vocal commands and had no signs of meningeal irritation. Cranial nerves, motor, sensory and cerebellar examination was unremarkable except for bilaterally up going plantars.

**Figure 1:**
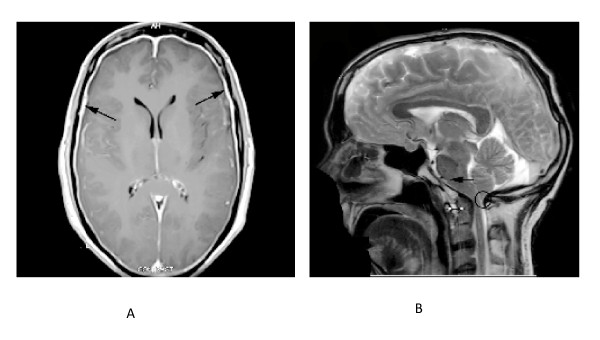
**MRI Brain**. **a**. Axial T1 weighted post contrast image; significant meningeal enhancement (black arrows) suggestive of pachymeningitis is shown. **b**. Sagittal T2 weighted image; showing characteristics of Spontaneous Intracranial Hypotension: flattening of pons (white arrow) and inferior orientation of cerebellar tonsils (encircled). Atlanto-axial subluxation (white double arrow), is also demonstrated.

**Figure 2:**
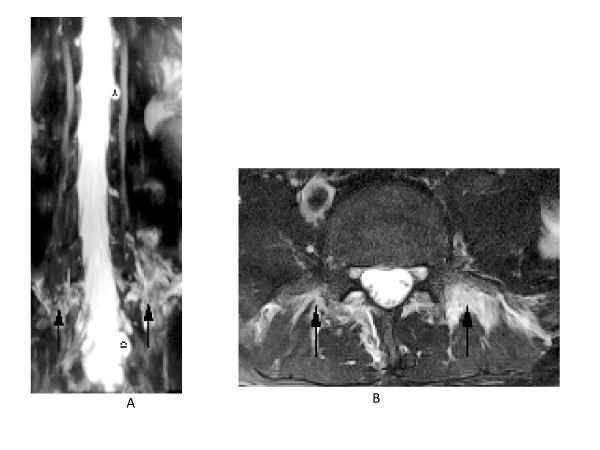
**a. Time of Flight MR Myelogram of Lumbosacral Spine revealing multiple dural out pouchings representing dural ectasias (black asterisks) and b MRI Lumbar spine**. **a**. Also seen is CSF fluid in para-spinal muscles (black arrows). **b. **T1 weighted post contrast Image, revealing leakage of Cerebrospinal fluid into the adjacent paraspinal musculature (white arrows) from dural ectasias (not shown here).

Routine blood tests were normal. MRI brain with contrast revealed pachymeningitis (Figure 1a), atlanto-axial subluxation, cerebellar tonsillar herniation and flattening of pontine surface (Figure 1b). Since the clinico-radiological findings were suggestive of spontaneous intracranial hypotension probably secondary to a dural tear, an MR myelogram study with intrathecal contrast was performed that demonstrated expansion of thecal sac, more marked in the lumbosacral region with multiple dilated out-pouchings in the sacral regions, S1-3, representing dural ectasias (Figure 2a). Accumulation of fluid in the posterior para-spinal muscles was also observed (Figure 2b), further strengthening the suspicion of CSF leaks.

An autologous epidural blood patch using 25 ml of blood was applied at L4/5 level. This procedure resulted in marked improvement and almost complete resolution of his symptoms over the next 96 hours. In the first 48 hours after the procedure, strict recumbent position was maintained. He was then discharged with instructions to gradually resume sitting posture from complete bed rest by adding one to two pillows under his head every day. This was continued until he was able to stand upright without headaches.

He was followed up regularly as an outpatient for the next 6 months with no evidence of any complications or recurrence and returned to normal baseline activity and lifestyle.

## Discussion

Spontaneous intracranial hypotension (SIH) is increasingly recognized as a clinically variable syndrome caused by non-traumatic CSF leaks. Connective tissue disorders (CTDs) like Marfan syndrome are the most important predisposing conditions.

There is only one case report in the literature that links spontaneous CSF leak complicated by tonsillar herniation in a Marfan patient [17]. This complication was also seen in our patient.

Tonsillar herniation is considered a part of the SIH complex and has been labeled as "acquired Chiari - Malformation I (CM-I)" [17]. Lack of awareness or erroneous diagnoses has been reported to lead to unnecessary surgical intervention such as suboccipital craniectomy for posterior fossa decompression in an attempt to treat intractable headaches thought to be caused by tonsillar herniation; only later was it realized that the real cause of headaches was SIH secondary to CSF leaks [17].

Amongst the major abnormalities detected on an MRI study, thickening of pachymeninges and engorgement of venous sinuses occur as a result of vascular dilation to compensate for the reduced CSF volume in accordance with the Monro-Kellie hypothesis [18].

A spectrum of treatment method exists to manage SIH. It is reported that bed rest relieves headache of ICH through reduced CSF pressure at the site of leakage allowing rapid healing of the meningeal defects [19]. Although widely accepted, this concept has been challenged by other investigators [20].

Attempts to increase CSF volume include oral and intravenous rehydration, increased salt intake and steroid therapy. Although there has been some clinical improvement in patients with these modes of therapies, conclusive studies proving efficacy are still awaited; in fact some studies have shown these measures to be of questionable use [21].

Epidural blood patches are generally considered a safe and effective option after bed rest and conservative management. It has been found that the procedure success rate is higher if it is performed at or within one interspace of the leak [22].

Surgical correction is required only when all the other measures have failed and a meningeal defect has been demonstrated. Simple ligation of the meningeal diverticula can achieve complete resolution of symptoms in 100% of patients [23].

## Conclusion

Spontaneous intracranial hypotension is now being recognized as a fairly rare entity but is more commonly seen in settings of connective tissue disorders. However, general physicians and neurologists should be aware of this condition while providing consultations to patients with postural headaches. Proper education to physicians regarding the diagnosis, evaluation and management of SIH can save patients from embarking on invasive surgical procedures.

## Abbreviations

SIH: Spontaneous intracranial hypotension; CTDs: connective tissue disorders; MRI: Magnetic Resonance Imaging; ICH: Intracranial Hypotension; CSF: Cerebrospinal Fluid.

## Competing interests

The authors declare that they have no competing interests.

## Authors' contributions

**AHP **conceptualized the study, collected patient data and drafted the manuscript. **FAM **collected patient data and drafted the manuscript. **NAS **and **HA **proofread the draft and provided expert opinion in contextualizing the data. All authors have read and approved the final manuscript.

## Pre-publication history

The pre-publication history for this paper can be accessed here:

http://www.biomedcentral.com/1471-2377/10/54/prepub

## References

[B1] Schaltenbrand V Neuere Anschauungen zur Pathophysiologie der Liquorzirkulation Zbl Neurochir 1938 3 290 295

[B2] Rando TA Fishman RA Spontaneous intracranial hypotension: report of two cases and review of the literature Neurology 1992 42 481 487 1549206 10.1212/wnl.42.3.481

[B3] Schievink WI Meyer FB Atkinson JLD Spontaneous spinal cerebrospinal fluid leaks and intracranial hypotension J Neurosurg 1996 84 598 605 10.3171/jns.1996.84.4.0598 8613851

[B4] Schievink WI Roiter V Baumgartner RW, Bogouss-lavsky J, Caso V, et al Epidemiology of cervical artery dissection Handbook of Cerebral Artery Dissection 2005 Basel, Germany: Karger 12 15 full_text

[B5] Schievink WI Misdiagnosis of spontaneous intracranial hypotension Arch Neurol 2003 60 1713 1718 10.1001/archneur.60.12.1713 14676045

[B6] Fukutake T Sakakibara R Mori M Araki M Hattori T Chronic intractable headache in a patient with Marfan's syndrome Headache 1997 37 291 305 10.1046/j.1526-4610.1997.3705291.x 9195769

[B7] Rosser T Finkel J Vezina G Majid M Postural headache in a child with Marfan syndrome: case report and review of the literature J Child Neurol 2005 20 153 155 10.1177/08830738050200021701 15794185

[B8] Milledge JT Ades LC Cooper MG Jaumees A Onikul E Severe spontaneous intracranial hypotension and Marfan syndrome in an adolescent J Paediatr Child Health 2005 41 68 71 10.1111/j.1440-1754.2005.00541.x 15670230

[B9] Schievink WI Gordon OK Tourje J Connective tissue disorders with spontaneous spinal cerebrospinal fluid leaks and intracranial hypotension: a prospective study Neurosurgery 2004 54 65 70 10.1227/01.NEU.0000097200.18478.7B 14683542

[B10] Schievink WI Torres VE Spinal meningeal diverticula in autosomal dominant polycystic kidney disease Lancet 1997 349 1223 1224 10.1016/S0140-6736(05)62417-8 9130952

[B11] Capobianco DJ Kuczler FJ Jr Case report: primary intracranial hypotension Milit Med 1990 155 64 66 2106653

[B12] Lipman IJ Primary intracranial hypotension. The syndrome of spontaneous low cerebrospinal fluid pressure with traction headache Dis Nerv Syst 1977 38 212 213 837825

[B13] Teng P Papatheodorou C Primary cerebrospinal fluid hypotension Bull LA Neurol Soc 1968 33 121 128 5669496

[B14] Garcia-Albea E Cabrera F Tejeiro J Delayed postexertional headache, intracranial hypotension and racket sports J Neurol Neurosurg Psychiatry 1992 55 975 Letter 10.1136/jnnp.55.10.975 1431965 PMC1015204

[B15] Horton JC Fishman RA Neurovisual findings in the syndrome of spontaneous intracranial hypotension from dural cerebrospinal fluid leak Ophthalmology 1994 101 244 251 8115145 10.1016/s0161-6420(94)31340-6

[B16] Atkinson JL Weinshenker BG Miller GM Piepgras DG Mokri B Acquired Chiari I malformation secondary to spontaneous spinal cerebrospinal fluid leakage and chronic intracranial hypotension syndrome in seven cases J Neurosurg 1998 88 237 242 10.3171/jns.1998.88.2.0237 9452230

[B17] Puget S Kondageski C Wray A Boddaert N Roujeau T Di Rocco F Zerah M Sainte-Rose C Chiari-like tonsillar herniation associated with intracranial hypotension in Marfan syndrome J Neurosurg 2007 106 1 Suppl 48 52 Case report 17233313 10.3171/ped.2007.106.1.48

[B18] Fishman RA Dillon WP Dural enhancement and cerebral displacement secondary to intracranial hypotension Neurology 1993 43 609 611 8451008 10.1212/wnl.43.3_part_1.609

[B19] Nosik WA Intracranial hypotension secondary to lumbar nerve sleeve tear JAMA 1955 157 1110 1111 10.1001/jama.1955.02950300038008 14353648

[B20] Teece S Crawford I Bed rest after lumbar puncture Emerg Med J 2002 19 432 3 10.1136/emj.19.5.432 12204997 PMC1725971

[B21] Mokri B Piepgras DG Miller GM Syndrome of orthostatic headaches and diffuse pachymeningeal gadolinium enhancement Mayo Clin Proc 1997 72 400 413 10.4065/72.5.400 9146681

[B22] Davenport RJ Chataway SJ Warlow CP Spontaneous intracranial hypotension from a CSF leak in a patient with Marfan's syndrome J Neurol Neurosurg Psychiatry 1995 59 516 519 10.1136/jnnp.59.5.516 8530937 PMC1073715

[B23] Lay C Campbell K Mokri B Goadsby PJ, Silberstein SD Low cerebrospinal fluid headache Headache 1997 Boston: Butterworth-Heinemann 355 367

